# p16 Immunohistochemical Expression in Nephrogenic Adenoma

**DOI:** 10.7759/cureus.41285

**Published:** 2023-07-02

**Authors:** Juan Carlos Alvarez Moreno, Hafiz A Ghani, Vasily Ovechko, Cecilia Clement, Eduardo Eyzaguirre

**Affiliations:** 1 Pathology, University of Texas Medical Branch at Galveston, Galveston, USA

**Keywords:** urothelial carcinoma, clear cell carcinoma, genitourinary tract, p16, nephrogenic adenoma

## Abstract

Nephrogenic adenoma (NA) is a rare metaplastic entity commonly associated with a prior urothelial injury. Most are seen in the urinary bladder and a minority involve the urethra. In this study, we evaluated the expression of p16 as a surrogate marker of this entity and correlated it with clinical pathological parameters. A total of 17 cases of NA were retrospectively studied to assess the immunohistochemical expression of p16 and its value for the diagnosis of this entity.

## Introduction

Nephrogenic adenoma (NA) in the genitourinary tract is a rare metaplastic benign entity [1]. The term was introduced by Friedman and Kuhlenbeck because it resembled a renal tubule [2]. Microscopically, it consists of tubules, and papillary fronds lined by cuboidal eosinophilic cells or flat epithelium with hobnail nuclei. Nuclear enlargement with hyperchromasia and prominent nucleoli can be present, although frequently focal and of degenerative nature. These atypical features can cause possible confusion with prostate cancer and with clear cell adenocarcinoma of the urethra [2]. According to the literature, it has been associated with recurrent urinary tract calculi, recurrent urinary tract infections, trauma, prior surgery, diverticula of the urinary bladder, intravesical therapy, kidney transplantation, radiotherapy, foreign bodies, and chemical agents [3]. The most common location is the urinary bladder (80%) and other locations include the urethra (15%), ureter (5%), and rarely the renal pelvis [3].

NA can express PAX8 as a renal transcription factor that is always co-expressed with PAX2 in embryonic and adult renal tissues. These are cell lineage-restricted transcription factors expressed in normal and neoplastic tissues of renal tubular cells in fetal and adult kidneys [4]. The INK4 class are cell cycle inhibitors with a tumor suppressor gene known as p16 (CDKN2A); therefore, p16 overexpression drives cell death and apoptosis [5]. Hence, some non-human papillomavirus (HPV) tumors (melanoma, mesothelioma, liposarcoma, serous carcinoma) can have p16 positive expressions. p16 expression can be seen in metaplastic and atrophic cells of cervical origin [6-7]. HPV expresses high levels of p16 [8]; however, there is no connection between p16 expression by immunohistochemistry and HPV infection [1]. To the best of our knowledge, there are no reports in the literature regarding p16 expression in NA. Here, we explore p16 expression in NA, with attention to clinical and pathologic characteristics, and assess the potential pitfalls associated with the use of this marker.

## Materials and methods

**Figure 1:**
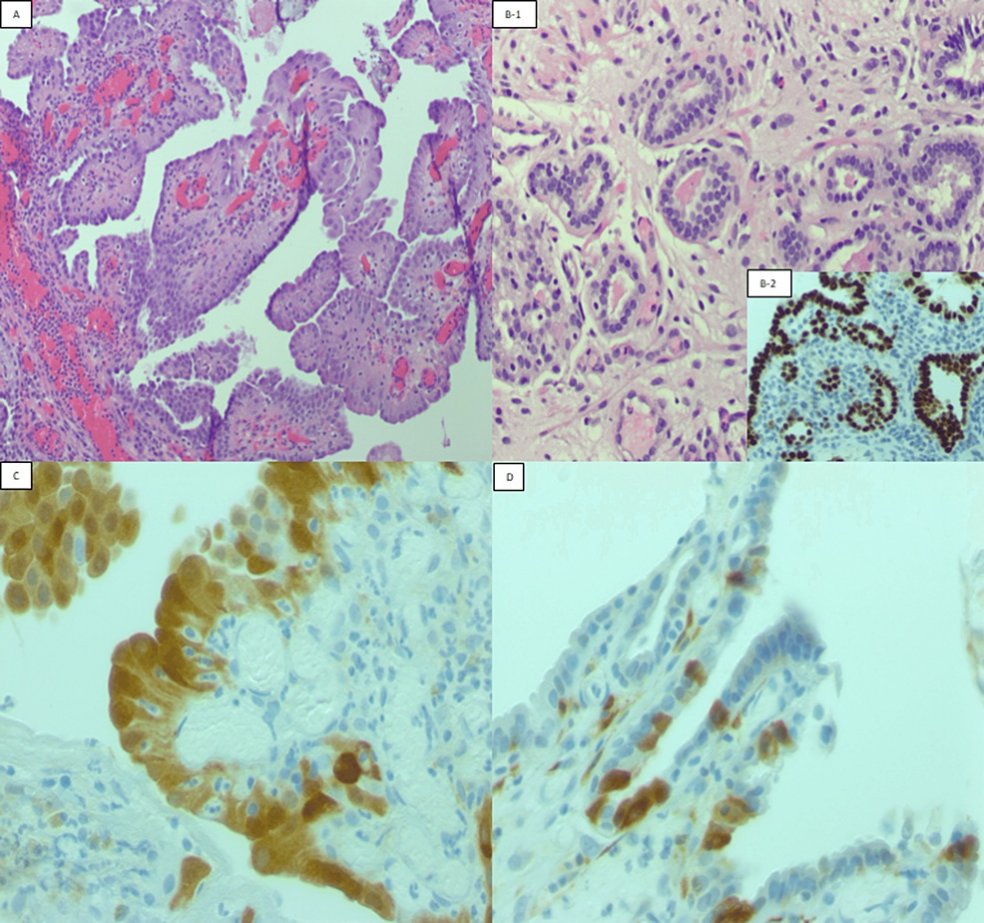
(A) H&E: 20X Papillary fronds lined by cuboidal eosinophilic cells; (B) H&E: 1. 20x Vascular-like tubules lined by cuboidal epithelium, bland round nuclei with inconspicuous nucleoli, with eosinophilic luminal secretions, 2. 20x PAX-8 positive nuclear stain; (C) IHC: p16 en-bloc positive expression with nuclear and cytoplasmic staining; (D) IHC: p16 patchy positive expression with nuclear and/or cytoplasmic staining IHC: immunohistochemistry; H&E: hematoxylin and eosin

This retrospective study included 17 patients with NA from the University of Texas Medical Branch (UTMB), Galveston, Texas, United States. The collection period was from 2015 to 2022. We included patients with NA (Figure 1, A and B-1) from different locations, PAX-8 positive to support the diagnosis (Figure 1, B-2), and a p16 immunohistochemical (IHC) stain was performed. For the patients who met the inclusion criteria, their clinical information was redeemed from medical records, including age, HPV status, gender, history of transplant or malignancy, and urolithiasis.

We took sections from tissue blocks embedded in paraffin and slides were stained with p16 primary antibody (Clone CINtec 16, Ventana). The corresponding hematoxylin & eosin (H&E) slides were analyzed in accordance with their compatible IHC stains. The IHC stain was positive when brown-gold cytoplasmic and/or nuclear staining and negative with no expression. p16 expression was categorized into en-bloc and patchy patterns (Figure 1, C and D).

## Results

The clinicopathological features are given in Table 1. The mean age was similar in both groups of p16 pattern. The en bloc pattern had more cases of NA than the patchy pattern. The male population showed slightly more cases of NA, and was predominant in both p16 patterns. A few cases in both groups had a history of bladder cancer. The en bloc pattern group had a higher history of prostate cancer than the patchy pattern group. There were few cases with calculi in both groups. The most common lesion type in the en bloc pattern group was erythema, while in the patchy pattern group were polyps. The patchy pattern group showed more cases being single lesions rather than multiple compared to the en bloc group. Cystourethroscopy was the most common procedure in both groups. The most common location identified in both groups was lateral in the bladder as well as posterior for the patchy group. Only one HPV-positive case was present in each group. 

**Table 1:** Clinicopathological Factors HPV: human papillomavirus

Clinicopathological Factors	En bloc (n=10)	Patchy (n=7)
Age (years, mean ± standard deviation)	57.1 ± 17.5	58.9 ± 18.9
Gender, n(%)		
Female	4 (40.0)	2 (28.6)
Male	6 (60.0)	5 (71.4)
History of bladder cancer, n(%)		
No	9 (90.0)	5 (71.4)
Yes	1 (10.0)	2 (28.6)
History of prostate cancer, n(%)		
No	1 (10.0)	3 (42.8)
Yes	8 (80.0)	2 (28.6)
Not applicable	1 (10.0)	2 (28.6)
Calculi, n(%)		
No	8 (80.0)	5 (71.4)
Yes	2 (20.0)	2 (28.6)
Lesion type, n(%)		
Polyp	1 (10.0)	3 (42.8)
Erythema	3 (30.0)	2 (28.6)
Sessile	1 (10.0)	2 (28.6)
Papillary	2 (20.0)	0 (0.0)
Stricture	1 (10.0)	0 (0.0)
Diverticulum	1 (10.0)	0 (0.0)
No data	1 (10.0)	0 (0.0)
Number of lesions, n(%)		
Single	4 (40.0)	6 (85.7)
Multiple	3 (30.0)	1 (14.3)
No data	3 (30.0)	0 (0.0)
Location, n(%)		
Bladder, lateral	3 (30.0)	3 (42.8)
Bladder, posterior	0 (0.0)	4 (57.2)
Bladder, other	5 (50.0)	0 (0.0)
Prostate	1 (10.0)	0 (0.0)
Urethra	1 (10.0)	0 (0.0)
Procedure, n(%)		
Cystourethroscopy	6 (60.0)	6 (85.7)
Transurethral resection of prostate	1 (10.0)	1 (14.3)
Transurethral resection of bladder tumor	2 (20.0)	0 (0.0)
Hysterectomy	1 (10.0)	0 (0.0)
HPV status, n(%)		
Positive	1 (10.0)	1 (14.3)
Negative	2 (20.0)	0 (0.0)
No data	7 (70.0)	6 (85.7)

## Discussion

We found that p16 nuclear and cytoplasmic expression was positive in all NA cases. The pattern distribution in 10 cases was en bloc and in seven cases was patchy. In a study by Tringler et al p16 positivity in the uterine cervix was seen in 6.5% of normal squamous mucosa, 37.5% in ciliated columnar cells of endocervical glands, 30% of Nabothian cysts, and 100% in tubal metaplasia [9]. In the oropharynx, there is evidence of focal p16 expression in benign tonsillar tissue, nondysplastic squamous epithelium, tumor stroma, and benign papillomas [10,11]. Most of these cases showed a mosaic pattern of distribution rather than en bloc. Our study is the first to acknowledge this pattern of expression. In anogenital lesions, the p16 protein expression should be nuclear or nuclear and cytoplasmic when associated with HPV [12]. Our cohort showed both patterns as previously mentioned.

NAs can present as irritative voiding symptoms and the usual complaint is hematuria [13]. Imaging can show polypoid or sessile masses within the bladder with a very nonspecific appearance [14]. Gross examination can reveal papillary, polypod, fungating, or sessile lesions [15]. As mentioned previously, they can have cysts, tubule formation, and solid and papillary growth [16]. Tubules are surrounded by thickened hyalinized membranes and lined by hobnail, cuboidal, or low columnar cells [16]. Their IHC profile shows positive stains for PAX8, BerEP4, and S100A1, and negative for PSA, CEA, and P63 [16].

The main differential diagnosis for this entity is clear cell carcinoma (Table 2). They can also present with hematuria, dysuria, urinary urgency, and recurrent UTIs. They can have NA hobnailed nuclei, vascular-like tubules with attenuated epithelium, and eosinophilic cells with different architectural patterns of growth, such as papillary, tubulocystic, or solid [17]. The most predominant features are clear cells, necrosis, severe atypia, high Ki-67, and mitotic rate [17]. By imaging, they can present as hydronephrosis or hydroureter, and grossly they present as solid, papillary, sessile, polypoid, and fungating lesions like NAs [17]. They can also be positive for cytokeratins (CK20, CK7), CEA, Napsin A, PAX8, and CA125 and CD10 is negative in these tumors [17]. In clear cell carcinomas, p16 can show a diffuse pattern [17]. This falls into the pitfall with nephrogenic adenomas with our current results.

**Table 2:** Differential Diagnosis between Nephrogenic Adenoma and Clear Cell Carcinoma CK: cytokeratin; CEA: carcinoembryogenic antigen; CD10: cluster of differentiation 10; P63: tumor protein 63; BerEP4/EpCAM: Epithelial Cell Adhesion Molecular; Pax-8: paired box gene 8; UTI: urinary tract infection; PSA: prostatic-specific antigen; CA125: cancer antigen 125

	Nephrogenic Adenoma	Clear Cell Carcinoma
Symptoms	Palpable mass, recurrent UTIs, obstructive urinary symptoms, hematuria	Hematuria, dysuria, obstructive urinary symptoms, recurrent UTIs
Imaging	Solid or sessile masses	Solid mass, hydronephrosis, or hydroureter
Gross appearance	Solid, sessile, papillary, fungating	Solid, papillary, sessile, polypoid, fungating
Nuclear atypia	Rare	Common
Histological features	Tubules surrounded by hyalinized basement membrane	Tubulocystic pattern with hobnail and clear cells
Immunohistochemistry	Positive PAX8, S100A1, BerEP4/EpCAM, Negative P63, PSA, CEA	Positive CK7, CK20, CA125, CEA; Negative CD10

Another entity in the differential is invasive urothelial carcinoma. They present with gross or microscopic hematuria, urinary urgency and frequency, and dysuria [18]. Systemic symptoms can manifest with weight loss, fatigue, bone pain, and metastasis to lymph nodes, liver, lung, and bone [19,20]. The most common location is the bladder (90%) and the upper urinary tract (renal pelvis and/or ureter) accounts for 5-10% [21]. The macroscopic examination can be papillary, sessile, polypoid, nodular, and ulcerative, also unifocal or multifocal [21]. The histologic variants are multiple including conventional, squamous, glandular, trophoblastic, nested, microcystic, micropapillary, lymphoepithelioma-like, plasmacytoid/signet ring/diffuse, sarcomatoid, giant cell, lipid-rich, clear cell, and poorly differentiated [22]. The IHC markers that support the urothelial lineage are GATA3, p63, and high-molecular-weight cytokeratins [23]. p16 can be positive in these tumors; therefore, it has limited utility in distinguishing between cervical squamous cell carcinoma and primary squamous cell carcinoma of the bladder [24]. In a study by Hashmi et al., they classified the p16 IHC stain as low expression and high expression [25]. The high-expression group showed worse long-term survival.

The third entity in the differential is the prostatic acinar adenocarcinoma. This cancer is asymptomatic and detected by increased levels of serum prostatic-specific antigen (PSA) [26]. It can also be incidental or present with hematuria [27]. The primary diagnostic imaging is transrectal ultrasound and MRI [28]. The most common site of distant metastasis is the bone [29]. These tumors are not clearly visible macroscopically, and when they are, they are firm, solid, and poorly circumscribed [30] and are most commonly located in the peripheral zone [31]. Microscopically, the major criteria are haphazard infiltration, nuclear atypia, and absence of basal cells [32]. You can also have more complex architecture, crowding, cords, and fused and cribriform glands [28]. Minor criteria include amphophilic cytoplasm, nuclear hyperchromasia, luminal amorphous eosinophilic material, and crystalloid and blue mucinous secretions [28]. The IHC stains for basal cell markers are high-molecular-weight cytokeratins like CK5/6 or p63, also for cytoplasmic stains we use AMACR [33]. In a study by Takahara et al., p16 expression was an adverse prognostic marker in high-grade prostatic adenocarcinomas [34].

Our study had limitations. First, the number of patients in our cohort was a small sample size from a single institution; a larger study population is warranted for greater generalizability and significance. Second, there was a lack of HPV testing on the majority of our patients, and could not provide a correlation between HPV and p16 expression. We did not compare a different p16 antibody clone to see a different pattern of expression. Finally, no other reports in the literature are available regarding the expression of p16 antibody in NA; therefore, we could not compare our results to others. It would be of interest for future studies to study the correlation between NA, p16, and HPV status. 

## Conclusions

The expression of p16 in nephrogenic adenoma is unique and new to the literature.The differential diagnosis includes clear cell carcinoma, urothelial carcinoma with p16 expression and prostatic adenocarcinoma. The correlation with HPV status is still unknown; therefore, it would be interesting if future studies and investigations did HPV molecular testing on these patients, especially in a bigger population.
